# Management of Lumbar Disc Herniation: A Systematic Review

**DOI:** 10.7759/cureus.47908

**Published:** 2023-10-29

**Authors:** Akram M Awadalla, Alaa S Aljulayfi, Abdulaziz R Alrowaili, Hatem Souror, Fay Alowid, Abdulaziz Mahdi M Mahdi, Remaz Hussain, Mujib M Alzahrani, Ahmad N Alsamarh, Esam A Alkhaldi, Reem C Alanazi

**Affiliations:** 1 Neurological Surgery, King Salman Armed Forces Hospital, Tabuk, SAU; 2 College of Medicine, King Saud Bin Abdulaziz University for Health Sciences, Riyadh, SAU; 3 Faculty of Medicine, University of Tabuk, Tabuk, SAU; 4 Medicine and Surgery, University of Jeddah, Jeddah, SAU; 5 Orthopedic Surgery, University of Jeddah, Jeddah , SAU; 6 Plastic and Reconstructive Surgery, Taibah University, Medina, SAU; 7 Faculty of Medicine, Al Baha University, Al Baha, SAU; 8 Faculty of Medicine, Al Jouf University, Sakaka, SAU; 9 Faculty of Medicine, King Khalid University, Abha, SAU; 10 Family Medicine, Primary Health Care Corporation, Riyadh, SAU

**Keywords:** back pain, surgical treatment, sciatica, ligamentum flavum, lumbar disc herniation

## Abstract

A herniated disc in the spine is a condition during which a nucleus pulposus is displaced from intervertebral space. It is a common cause of back pain. The patients who experience pain related to a herniated disc often remember an inciting event that caused their pain. This activity reviews the evaluation and management of lumbar disc herniation and discusses the role of the healthcare team in evaluating and improving care for patients with this condition. Data sources were PubMed/Medline and Embase. Our review investigated English-language articles (from 2010 to 2023) according to the PRISMA guidelines. Overall, there were seven articles. Surveys and analyses of national databases were the most widely used methods (n=7). The search identified 777 studies; 7 were eligible for inclusion in the analysis. Further understanding of spinal disc herniation and treatment protocols may help improve evaluation and management in the future. Our research covered a range of management options. Disc herniation is a frequent problem for internists, emergency department doctors, nurse practitioners, and primary care physicians. To manage efficiently, an interprofessional team is needed. The first course of treatment is conservative, with paracetamol and anti-inflammatories being frequently used to relieve pain. A chemist must supervise the use of opioid analgesics in certain situations. Although surgery is sometimes the final option, patients frequently have neurological damage and lingering discomfort. In circumstances where physical treatment is not working, MRI interpretation becomes necessary. Primary care physicians or mental health professionals should handle back pain as it is frequently linked to mental health issues. Results can be enhanced by regular exercise and preserving a healthy body weight.

## Introduction and background

Back discomfort is caused by a herniated disc in the spine, which is a condition in which the nucleus pulposus is displaced from the intervertebral space. Pain may radiate into the lower extremities, be searing or stinging, and be accompanied by changes in sensation or weakening. The disc supports the spine by serving as a cushion against trauma between the vertebral bodies. Pain may occasionally result from the disc compressing a nerve or spinal cord. Although herniated discs can cause pain, there aren't many efficient conservative therapy options. Although refractory instances may require surgical repair or interventional techniques, most cases heal conservatively. When a patient has a herniated disc, the healthcare provider should keep an eye out for any serious neurological abnormalities that can require an immediate referral for neurosurgery [1-3].

When the nucleus pulposus pushes through the annulus fibrous, a thick collagenous ring that surrounds the nucleus pulposus, disc herniation happens. As people age, a degenerative process weakens the nucleus pulposus, which is the most common reason and results in worsening symptoms. Disc herniation is also a result of congenital abnormalities, connective tissue diseases, and trauma. Because of biomechanical pressures, it occurs more frequently in the lumbar and cervical spines, whereas the thoracic spine has a lesser incidence [4].

Localized inflammation and mechanical compression of the nerve by the protruding nucleus pulposus are the causes of herniated discs. The nerve root is compressed in posterior lateral herniations because the annulus fibrosus is thinner and there is less structural support. Clinical myelopathy and spinal cord compression can result from large midline disc herniations. The combination of chemical irritation brought on by inflammation and disc pressure on the longitudinal ligament results in localized back discomfort [2]. Adults with a 2:1 male-to-female ratio who are 3 to 5 years old are prone to herniated discs [5].

Herniated discs can cause pain, tingling, numbness, diminished feeling, weakening, and instability. The most frequent disc in the cervical spine, the C6-7, is the source of radiculopathy. The focus of a physical examination should be on deficiencies and the distribution of sensory abnormalities. The lower region of the thoracic spine is affected by thoracic discogenic pain syndrome, which is frequently brought on by thoracic disc degeneration. The majority of thoracic disc herniations are asymptomatic and are unintentionally found during an MRI. Paralysis, abnormalities of the cardiovascular system, and alterations of gait are examples of serious findings. Herniated discs in the lumbar spine may cause impairments in motor function and sensation that are exclusive to a particular myotome. Localizing the level of compression and determining the sensory loss, weakness, pain location, and reflex loss associated with varying levels can be facilitated by a neurological examination [6-8].

Non-surgical interventions, such as non-steroidal anti-inflammatory drugs (NSAIDs) and physical therapy, are the mainstay of management for acute cervical and lumbar radiculopathies caused by a herniated disc. These work well for controlling incapacitating pain, but patients who are neurologically impaired or who do not improve with conservative care require prompt surgical consultation. There is little evidence to support the use of oral corticosteroids or muscle relaxants such as cyclobenzaprine; instead, opioid analgesics are recommended for severe pain that does not improve with over-the-counter painkillers. Second-line treatments for individuals whose symptoms have persisted for at least four to six weeks and who are not responding to conservative care include translaminar epidural injections and selective nerve root blocks [9,10].

The last option for treating herniated discs is surgery, which includes discectomies, laminectomies, artificial disc replacement, anterior cervical decompression and fusion, and other lumbar spine procedures. The benefits are modest and usually wane over time. Complete discectomy and fusion are options. Surgical interventions typically have transient benefits [3]. Numerous disorders, including discal cyst, degenerative spinal stenosis, mechanical back pain, epidural abscess, hematoma, metastasis, diabetic amyotrophy, neurinoma, osteophytes, cauda equina syndrome, and synovial cyst, can be detected with herniated discs [11]. Radiofrequency ablation procedures or epidural steroid injections can be used to treat progressive myelopathic symptoms. Transthoracic or costotransversectomy techniques are surgical methods used for thoracic spine discectomies and fusions [12]. Primary care physicians or mental health professionals should handle back pain as it is frequently linked to mental health issues. Results can be enhanced by regular exercise and preserving a healthy body weight [13].

The aim of the spinal disc herniation review is to discuss its management to alleviate symptoms and avoid complications including lifestyle changes, pharmacological care like medications, and surgical treatments.

## Review

Methods

According to the Preferred Reporting Items for Systematic Reviews and Meta-Analyses (PRISMA) standards, the procedures for the current systemic were developed. 

Search

**Figure 1:**
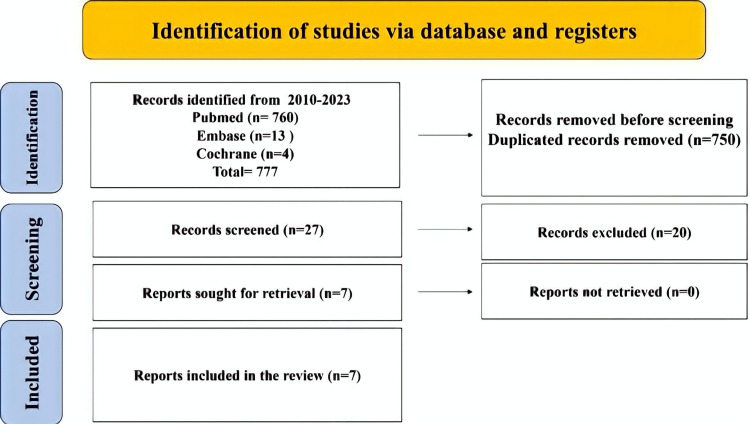
Study flow diagram using PRISMA PRISMA: Preferred Reporting Items for Systematic Reviews and Meta-Analyses

For this review, keywords and Medical Subjects Headings phrases were used to search PubMed/MEDLINE (2010-August 2023) and Embase (2010-August 2023) for three important concepts: Lumbar disc herniation, ligamentum flavum, sciatica, and surgical treatment. Limits were applied so that only English-language items were included. The supplementary material contains a complete description of the PubMed search methodology. Examining the references found in the identified papers led to the creation of additional studies. After retrieving all full texts, seven articles were discovered to be part of this review. The PRISMA technique was used for search screening and article shortlisting (Figure 1).

Inclusion criteria

English articles and articles and reviews demonstrating herniation, ligamentum flavum, sciatica, and surgical treatment were considered as inclusion criteria.

Exclusion criteria

Incomplete data, research data before 2010, and data that did not discuss spinal disc herniation were excluded.

Data extraction

Twenty-seven papers were included after titles and abstracts were checked out of the 777 studies that were found through the search. Seven papers were qualified for analysis after full-text reading (Figure 1). 

Data extraction

Table 1 provides a brief description of the data, which includes the author(s) name and year, the study aim, and finally the conclusion of the study.

**Table 1:** Brief description of the data SLDH: Symptomatic lumbar disc herniation

Serial no.	Author and year	Aim of the study	Conclusion
1	Wang et al.; 2020 [14]	The incidence of regression after the non-surgical treatment of symptomatic lumbar disc herniation: a systematic review and meta-analysis	Wang present a total IR of 63% for SLDH patients who were not surgically treated, giving clinical decision makers quantifiable proof of IR. When determining whether to undertake surgery for SLDH, we recommend a follow-up timeline with time points 4 and 10.5 months following onset, based on our systematic review.
2	Yoon et al.; 2021 [15]	Herniated discs: when is surgery necessary?	The indications for surgery for varying degrees of disc herniations are described in this review. Surgery is recommended for cervical disc herniation (CDH) if symptoms don't go away after six months and don't respond to conservative measures. Surgery for thoracic disc herniation (TDH) is recommended if non-surgical treatment is ineffective or exacerbates neurological symptoms. If MRI verifies the lumbar disc herniation (LDH) and there is no improvement after six weeks of conservative therapy, surgery is recommended.
3	Ali et al.; 2013 [16]	Lumbar disc herniation in patients with chronic backache	Seventy-nine percent of 477 individuals with chronic low back pain had strong radiographic evidence of disc prolapse at the lumbar vertebral levels. 52.6%, 39.5%, and 2.6% of these patients had disc herniation at L5-S1, L4-L5, and L3-L4. L1-L2 disc prolapse patients were not discovered. The study found that middle-aged individuals, particularly those with lower back pain, were more likely to have intervertebral lumbar disc prolapsed illness.
4	Arts al.; 2019 [17]	For lumbar disc herniation patients who are not responding to initial conservative treatment, the trial contrasts lumbar discectomy with bone-anchored annular closure, lumbar discectomy, and continued conservative care.	Lumbar disc herniation (LD) is more beneficial than conservative therapy (CC) in relieving back pain and leg pain, according to an analysis of 14 studies involving 3947 patients. Additionally, LD + AC was more successful in lowering the chance of reherniation and reoperation. Although the treatment impact was less in randomized studies, there was indirect evidence that LD + AC was superior to CC in lowering back pain, leg pain, and disability. According to the study's findings, LD is superior to CC in treating lumbar disc herniation symptoms that are resistant to first-line conservative treatment.
5	Lurie et al.; 2014 [18]	To assess the 8-year outcomes of surgery vs. non-operative care.	Individuals with a lumbar disc herniation who were carefully chosen and had surgery had better results than individuals who did not receive surgery; after four to eight years, there was little to no decline in either group's outcomes. Low-quality evidence suggested that surgical treatment is more effective than non-operative treatment in improving physical functions; no significant difference was observed in adverse events. No firm recommendation can be made due to instability of the summarized data.
6	Jacobs et al.; 2011 [19]	A comprehensive analysis comparing conservative treatment with surgery for sciatica caused by a ruptured disc in the lumbar region	The purpose of this study was to compare the efficacy of conservative treatment versus surgery for patients with sciatica brought on by lumbar disc herniations. Five RCTs were carried out to assess pain, functional status, perceived recovery, and missed workdays. There were two studies that showed little chance of bias. The outcomes demonstrated that, in contrast to extended conservative treatment, early surgery resulted in faster pain alleviation in individuals experiencing radicular pain for six to twelve weeks. However, after a year and a half, no discernible changes were discovered. Subsequent research ought to assess who gains most from conservative therapy versus surgery. Because of inadequate reporting and clinical heterogeneity, the data was not pooled.
7	Dan-Azumi et al.; 2018 [20]	A systematic study and meta-analysis comparing surgery and conservative treatment for lumbar disc herniation with radiculopathy	In summary, the available data indicates that early surgery, without any long-term differences, is preferable to conservative therapy for patients with lumbar disc herniation and radiculopathy. The populations of the included studies were highly diverse, so care should be taken when interpreting the review's findings.

Discussion

This review highlights spinal disc herniation for finding the more appropriate management options. This systematic review identified seven studies presenting spinal disc herniation. Gencay-Can et al. in 2010 demonstrated that for patients having their first microdiscectomy, an urgent rehabilitation program is advised. Combining exercise and cognitive intervention with positive reinforcement is a successful therapy strategy. Even in patients who had LDH surgery and experienced a return of symptoms following the initial procedure, it is being explored as a potential substitute for spinal fusion. In most cases, early postoperative activity yields great results with no problems. For these therapies to be considered beneficial in routine clinical practice, more clinical trials on the subject with higher methodological quality and numbers are required [21].

Khorami et al. in 2021 evaluated the methodological quality of international clinical practice guidelines on lumbosacral radicular pain (LRP), this study set out to identify them. A comprehensive exploration was carried out across multiple databases, such as MEDLINE, PEDro, and Google Scholar. There were four categories for the guidelines: "should do," "could do," "do not do," and "uncertain." The quality of the twenty-three guidelines varied (AGREE II total evaluation ranged from 17% to 92%). Straight leg raises tests, pain distribution mapping, gait analysis, and congruence of signs and symptoms are among the consistent diagnostic suggestions. Physical exercise, educational support, and discectomy are examples of therapeutic options. When conservative therapy is ineffective or steppage gait is evident, a referral to a specialist is advised [22].

Schünemann et al. devised the "GRADE-ADOLOPMENT" methodology, a technique for swiftly and effectively adapting current guidelines for guideline creation. Using evidence, this method enables developers to provide recommendations that are specific to the situation at hand. For instance, the UK comprehensive guideline (NICE) was modified into the Belgian guideline KCE for the country's citizens. A multidisciplinary procedure for developing a single evidence-based clinical practice guideline for individuals with low back pain was detailed by Harstall et al. [23,24].

Physical examinations, including SLR and crossed SLR tests, muscular and sensory testing, and reflex tests, are advised in the guidelines for the diagnosis of lumbar radicular pain. Nonetheless, the sensitivity and specificity of these tests have been called into question. According to a systematic review, most tests perform poorly as a diagnostic tool when used alone, but when utilized in combination, improved outcomes can be achieved. Patients with LRP have demonstrated high diagnostic accuracy on the SLR and slump tests. Exercises and physical therapy are also advised by guidelines since they have been shown to be helpful in the conservative treatment of people with lumbar radicular pain [25].

There is inconsistency in the guidelines for LRP diagnosis and treatment. It is not advised to use non-invasive therapies including heat/cold therapy, traction, massage, acupuncture, bed rest, or therapeutic ultrasound. Two excellent guidelines suggest bed rest for a few days, whereas three standards do not encourage it at all, unless in extreme circumstances. Bed rest is not recommended in high-quality guidelines, which begs the question of how effective it is as a non-invasive therapeutic option. Finally, recommendations for pharmaceutical therapies are not always clear [26].

It is not unexpected that there are conflicting recommendations for patients with lumbar reflex pain (LRP), given the ongoing lack of solid evidence about their efficacy. It's possible that the 2014 placebo-controlled experiment contributed to the uneven usage of paracetamol. Paracetamol is recommended in guidelines issued before 2014; more recent guidelines are cautious. The 2015 NHG guideline continues to recommend paracetamol as the initial analgesic option. According to a 2016 Cochrane analysis, NSAIDs are not significantly effective at reducing pain during LRP treatment. But according to two guidelines published after 2016, NSAIDs should be considered. Further investigation is required to assess the impact of NSAIDs on LRP. Small effects were noted during short-term follow-up for epidural injections in invasive therapies, despite varied recommendations for these injections [27].

After twenty-three clinical practice guidelines for patients with LRP were obtained, the AGREE II tool showed that the overall quality of these guidelines ranged from low to good. According to these standards, physical examinations should include the SLR test, the crossing SLR test, a steppage gait evaluation, mapping pain distribution, and an agreement of signs and symptoms. Imaging is generally not advised for routine use and is only advised in certain situations. When conservative therapy fails or steppage gait is evident, the guidelines for treatment are to prescribe physical activity, offer educational care, and make a referral to a professional. To provide the best care possible, healthcare providers and systems around the world should heed these consistent suggestions [22].

Level II or moderate evidence was discovered in the systematic review and meta-analysis of percutaneous adhesiolysis/neurolysis in the treatment of lumbar disc herniation. The research comprised one RCT and five observational investigations, each with a follow-up of at least six months in one case and twelve months in the others. In the sole RCT by Gerdesmeyer et al., 90 patients were examined; 44 were assigned to the placebo group and 46 to the neurolysis group. The findings demonstrated that, in one year, 90% of patients in the lysis group and 35% of patients in the placebo group had improved their ODI by more than 50%. One thousand eight hundred and twenty-one patients participated in the observational trials, which evaluated the efficacy of percutaneous neurolysis in the treatment of lumbar disc herniation or persistent lumbar radiculopathy. Strong evidence is shown in the results from five observational studies and one high-quality RCT, with a significant percentage of patients following up for at least a year in four of the studies and six months in one [28].

The purpose of the systematic review and meta-analysis by Manchikanti et al. is to investigate the potential usefulness of neurolysis in the treatment of lumbar radicular pain or persistent, recalcitrant disc herniation. According to the study, the caudal approach which calls for larger volumes is thought to be the oldest and safest method for percutaneous neurolysis. Steroid injections are frequently used in conjunction with the sacral hiatus, which is the most widely used method. Numerous research studies have been conducted on the efficacy, safety, cost-utility, and patterns of utilization of epidural procedures; most of these investigations have concentrated on entrance through the sacral hiatus [29].

Disc herniation and chronic lumbar radiculopathy have been successfully treated with epidural injections utilizing caudal, interlaminar, and transforaminal techniques. Negative research has been discovered, nevertheless, including possible bias in interventional pain therapy. Comparative systematic review and meta-analysis by Manchikanti et al. produced different findings when they employed a methodology that didn't change active-controlled studies into placebo-controlled trials. The absence of several RCTs and large-scale observational studies with moderate quality are among the systematic review's limitations [30].

The study by Benzakour et al. discussed that it is still unknown what specifically caused intervertebral disc herniation. If the symptoms worsen after a period of conservative treatment or if there is an exacerbation of the neurological deficiency, surgical treatments may be recommended. After all, one of the most common spine surgery operations carried out by specialists or even some general surgeons with the necessary training are a discectomy [31].

The study by Gugliotta et al. in 2016 examined the efficaciousness of conservative and surgical management for lumbar disc herniation in patients with symptoms. Patients with lumbar disc herniation experienced quicker alleviation from back pain symptoms after surgery, but midterm and long-term follow-up results did not indicate an advantage over conservative treatment [32].

The study by Ju et al. in 2023 showed that there are two types of endoscopic lumbar surgery: a unilateral biportal approach and a comprehensive endoscopic interlaminar and transforaminal approach. We conducted a thorough analysis of the body of research on endoscopic spinal surgery side effects. The avoidance of difficulties was the main emphasis of this investigation. The most frequent side effects from endoscopic spinal surgery, regardless of the technique used, are perioperative hematoma and dural rupture. temporary dysesthesia, damage to the nerve roots, and recurrence. However, lumbar as well as cervical and thoracic spinal diseases like disc herniation, lumbar spinal stenosis, foraminal stenosis, and recurrent disc herniation can be safely and effectively treated with endoscopic spinal surgery, including full endoscopic transforaminal and interlaminar and unilateral biportal approaches [33].

The results of the study by Aljallad et al. in 2023 study showed that in patients with chronic lower back pain and radiculopathy caused by lumbar disc herniation, the likelihood of success of six-month outcomes was significantly influenced by younger age, less education, and better coronal radiographic lumbar spine alignment [34].

A 2023 study by Malik et al. revealed new surgical approaches that use minimally invasive lumbar decompression and the insertion of specialized equipment known as interspinous spacer devices. To manage pain related to spinal stenosis, many studies that provide a comparative analysis have shown that surgical intervention is more effective than non-surgical therapies [35].

Pediatric lumbar disc herniation has been shown by Obeidat et al. (2023) to be an uncommon but dangerous disorder that can cause children and adolescents to experience severe pain and incapacity. Trauma, repetitive motion, congenital anomalies of the spine, and some hereditary variables are risk factors for the syndrome. The patients in the cases that were presented all recovered well from the procedure and were able to resume their regular activities without experiencing any aftereffects from the minimally invasive discectomy and foraminotomy. For pediatric patients with lumbar disc herniation, the best results are achieved through early identification and adequate therapy. To gain a deeper understanding of the pathophysiology, risk factors, and best practices for treating this illness in children and adolescents, more study is required [36].

The study by Gurung et al. in 2023 in our neighborhood district hospital investigated the benefits of lumbar discectomies for patients' function and discomfort. Surgery postponed for more than six months did not result in significantly worse outcomes. It could be incorrect to define a TTS value in the absence of an increasing neurological disability [37].

## Conclusions

Disc herniation is a frequent problem for internists, emergency department doctors, nurse practitioners, and primary care physicians. To manage efficiently, an interprofessional team is needed. The first course of treatment is conservative, with paracetamol and anti-inflammatories being frequently used to relieve pain. A chemist must supervise the use of opioid analgesics in certain situations. Although surgery is sometimes the final option, patients frequently have neurological damage and lingering discomfort. In circumstances where physical treatment is not working, MRI interpretation becomes necessary. Primary care physicians or mental health professionals should handle back pain as it is frequently linked to mental health issues. Results can be enhanced by regular exercise and preserving a healthy body weight.
